# First principles calculation of interface interactions and photoelectric properties of ZnSe/SnSe heterostructure

**DOI:** 10.1371/journal.pone.0304032

**Published:** 2024-05-24

**Authors:** Yang-Yang Zhao, Si-Yuan Sheng

**Affiliations:** 1 Department of Basic Education, Criminal Investigation Police University of China, Shenyang, Liaoning, China; 2 Department of Physics, Shenyang University of Chemical Technology, Shenyang, Liaoning, China; Guru Ghasidas University Department of Pure & Applied Physics, INDIA

## Abstract

Heterostructure engineering is an effective technology to improve photo-electronic properties of two dimensional layered semiconductors. In this paper, based on first principles method, we studied the structure, stability, energy band, and optical properties of ZnSe/SnSe heterostructure change with film layer. Results show that all heterostructures are the type-II band arrangement, and the interlayer interaction is characterized by van der Waals. The electron concentration and charge density difference implies the electron (holes) transition from SnSe to monolayer ZnSe. By increasing the layer of SnSe films, the quantum effects are weakened leading to the band gap reduced, and eventually show metal properties. The optical properties also have obvious change, the excellent absorption ability of ZnSe/SnSe heterostructures mainly near the infrared spectroscopy. These works suggest that ZnSe/SnSe heterostructure has significant potential for future optoelectronic applications.

## Introduction

Interface interaction is an efficient solution to regulate structural and electrical properties in two dimensional (2D) semiconductor heterostructures [1]. The 2D semiconductor heterstructures can not only integrate intrinsic properties of every components, but also stimulate specific electronic and optical properties. They have been widely investigated in theoretical and experimental because of their excellent application in photodetectors [2, 3], thermoelectricity [4], and photocatalyst [5–7]. Recently, heterostructures with a stack of 2D layer material and films have been produced to improve photoelectric properties and stimulate electrons transfer [8, 9] between different interface. During the past decades, more and more research has been done to tune characters of 2D heterostructures. The review of Q. Su et al. concluded that constructing a haterojunction can intefrate the respective advantages and mitigate the drawbacks of each component, they discussed the design of 2D material-based heterojunction photocatalysts with different configurations [10]. The work of Zongpeng Wang et al. highlights the fundamental mechanism, designing strategy and recent achievements of different kinds of heterojunction based photocatalysts [11]. Cao et al. studied the strain introduced interface effect of *CsPbI*_3_/*SnS* heterojunction [12], and the absorption ability was obviously enhanced. The group of Liu et al. made a research on mechanical properties of 25 kind of heterostructures to indicate the electrons and holes transporting mechanism [13].

As one of the important Zn-based II-VI semiconductor with wide band gap, high light absorption and excellent photoelectric performance [14], zinc selenide (ZnSe) has been considered a suitable material for optoelectronic devices. Moreover, due to high excitation energy and excellent photoelectric performance, ZnSe is widely used in light-emitting devices, solar cells, and photodetectors [15]. ZnSe nanostructures can be synthesized by numbers of ways [16–18]. More importantly, the monolayer ZnSe usually has similar properties with bulk counterpart.

The indirect bandgap semiconductor tin selenium (SnSe) has a widely research in thermoelectric field within the past decades. SnSe is a typical layered material belongs to IV-VI compounds with narrow bandgap [19, 20]. Recently the nanostructures of semiconductor chalcogenides have attracted much attention due to their applications in optoelectronic devices. For IV-VI chalcogenides, most studies have focused on PbS and PbSe nanocrystals, because their band gaps can be adjusted in the infrared and visible spectra by changing the size of the nanocrystals. However, the synthesis and research of tin-based chalcogenides such as SnS, SnSe and SnTe are relatively few. Among the tin chalcogenides, tin selenide is an important narrow band gap semiconductor, which has a wide range of applications in solar cells, supercapacitors, infrared optoelectronic devices, storage switching devices and photocatalysis. Tin selenide materials mainly exist in two stoichiometric ways: tin selenide (SnSe) and tin diselenide (SnSe_2_). SnSe is a p-type semiconductor with a narrow band gap and It adopts an orthorhombic layered structure that is conducive to two-dimensional growth. However, SnSe_2_ is an n-type semiconductor with a layered structure. Sn atoms are sandwiched between two Se planes, and the connection of the two Se planes is completed by van der Waals interaction [21–23]. Under experimental conditions, Sn^2+^ is often oxidized to Sn^4+^, resulting in the final composition of SnSe_2_ rather than SnSe. Therefore, there are few studies on SnSe in the field of optoelectronics. With the advancement of technology, more and more theoretical and experimental results demonstrate outstanding properties of SnSe, such as appropriate bandgap, large absorption coefficient and excellent carrier mobility [19, 20, 24–27].

Despite both ZnSe and SnSe nano-film materials have become more and more popular recently, the properties of ZnSe/SnSe heterostructure are not fully studied yet. In this article, based on the structural characteristics of ZnSe and SnSe, we construct a heterojunction material composed of a single ZnSe substrate and a layered SnSe film, and studied the energy band and spectral properties of the material with first principles method. Firstly, the structures of pure 2D materials ZnSe and SnSe were discussed to lay solid foundation for heterojunction construction. Then the lattice mismatches, binding energy and potential energy of ZnSe/SnSe heterostructure were calculated to explain stability character. Furthermore, the energy band and optical properties were discussed. Finally, some conclusions were summarized in the last section of this paper.

## Calculating methods

In the present work, we use first principles calculation to study the properties of ZnSe/SnSe heterostructure. In this process the *ab* *initio* simulation package (abinit) [28] based on density functional theory were implemented. The norm-conserving pseudopotentials [29] were employed to describe interactions between cores and valence electrons. According to the exchange correlation functional described by generalized gradient approximation (GGA) of Perdew-Burker-Ernzerhof (PBE) [30], the structure and energy band of materials were discussed. The energy cutoff of wave function was set to 612.27*eV*. The lattice parameter of ZnSe 4.11 × 4.11 × 6.07 (*Å*) with K-point grid of 12 × 12 × 8, those parameter of SnSe are 4.10 × 4.10 × 12.08 (*Å* and 12 × 12 × 1, respectively. The heterostructure system was constructed by vesta with supercell of 3 × 3 × 1 and K points of 8 × 8 × 2. The ground state energy of system was calculated with the guiding of absolute differences of total energy, and the convergence criterion of energy in self-consistency process was set to 1.0 × 10^−8^*hartree*. In order to explore optical properties, the density functional perturbation theory (DFPT) was introduced. Meanwhile, the characters of energy band, density of states, and optical spectrum were carried out using non-self-consistent calculation of tolerance on wavefunction squared residual with the energy convergence precision 1.0 × 10^−18^.

Although the van der Waals force is a weak intermolecular force, its influence on the physical properties of materials cannot be completely ignored. Since the vdW force is essentially a nonlocal correlation effect, the local and semi-local traditional density functionals cannot clearly express the vdW interaction. In this paper, we take vdW-DF2 to modify the exchange correlation term. The modified energy is expressed as follows: *E* = *E*_*K*−*S*_ + *E*_*vdW*_, and E_*K*−*S*_ is the energy of traditional Kohn-Sham equation, E_*vdW*_ is correction term describing van der Waals interaction [31].

## Results and discussion

### Structure 2D materials of ZnSe and SnSe

**Fig 1:**
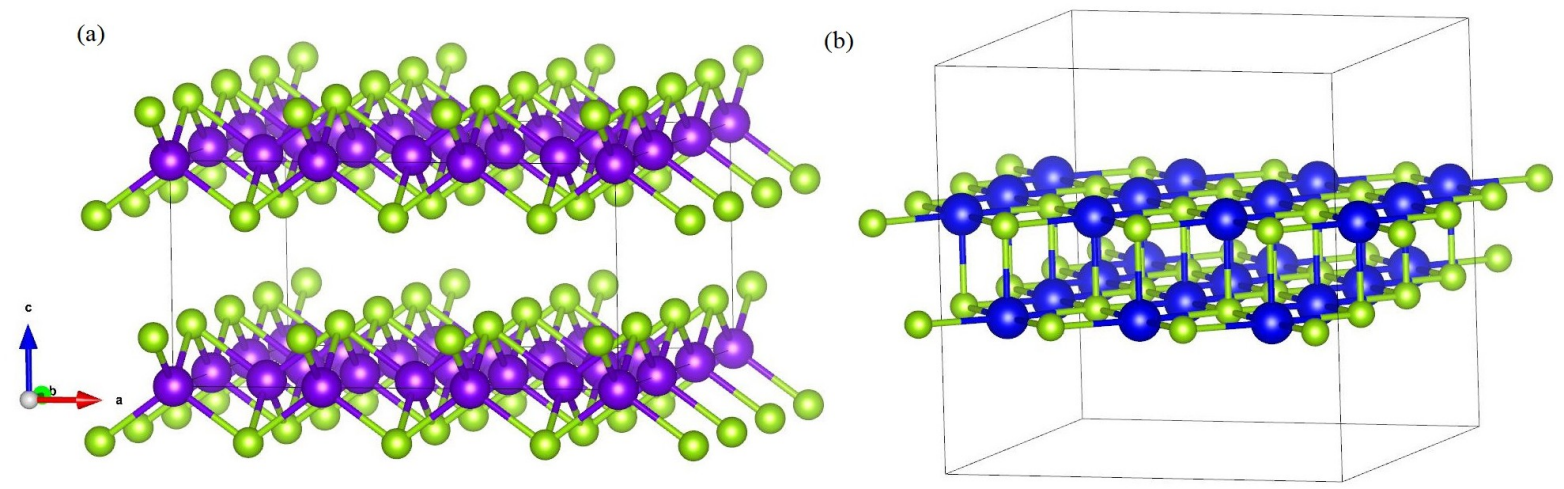
Side views structure of ZnSe and SnSe. Figure(a)-(b) show the geometric structures of 2D layered semiconductor materials ZnSe and SnSe, respectively. The purple and blue balls stand for Zn and Sn atoms, respectively; the little green ball stands for Se atom.

**Fig 2:**
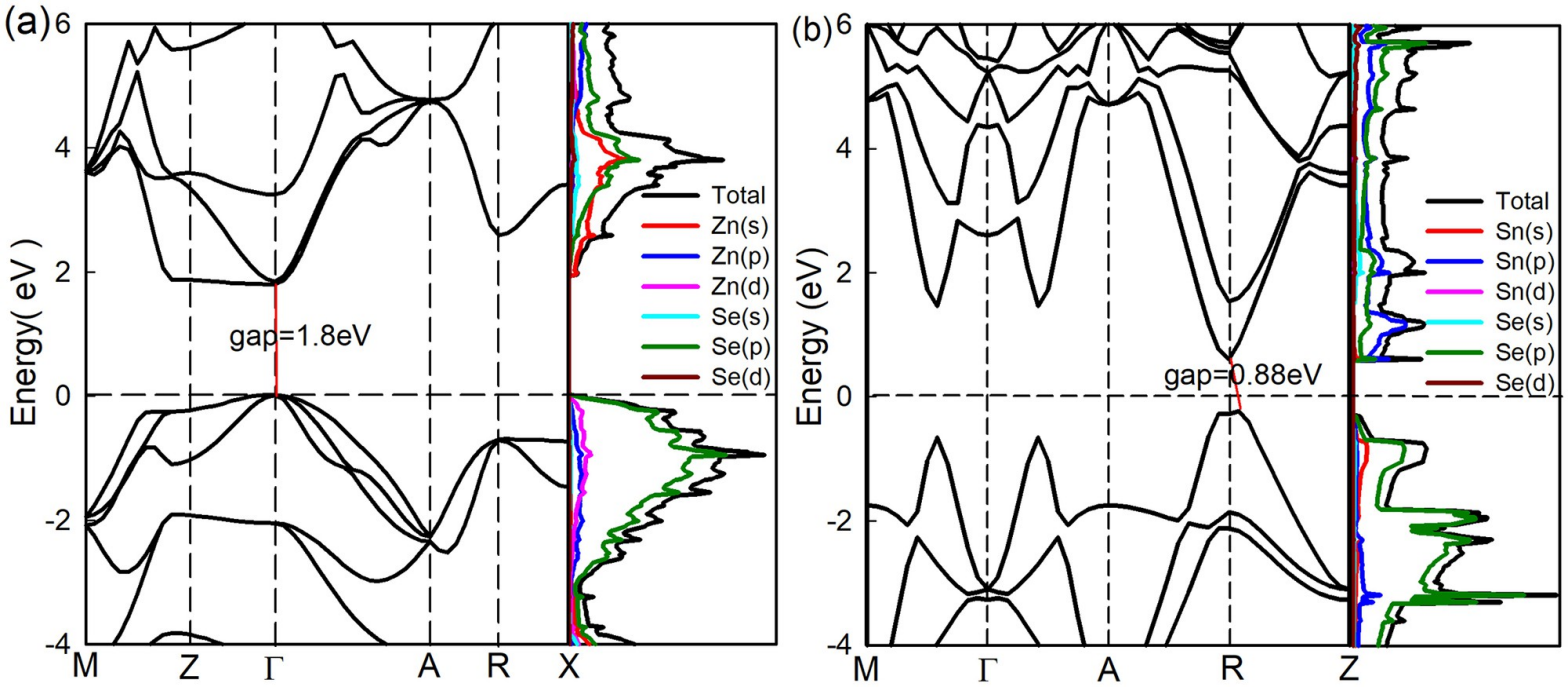
The band structure and density of states of 2D semiconductor ZnSe (a) and SnSe (b).

Both ZnSe and SnSe are 2D layered semiconductor materials, crystal structures of them along *z*-axis are decpicted in Fig 1. From Fig 1(a), we can see that Zn(Se) atom is bonded to its four nearest neighboring equivalent Se(Zn) atoms to form tetrahedral structure in a unit cell. While the week van der Waals combination between interlayer shows the characteristics of thin film materials. The calculated electronic band structure diagram of ZnSe is shown in Fig 2(a). The bottom of conduction band (CBM) and top of valence band (VBM) reside at *Γ* point in the Brillouin zone with a direct band gap of 1.80*eV*. The conduction band is contributed by hybridized orbitals of Se-3p and Zn-4s states, while the valence band is mainly contributed by Se-3p electrons and partly of Zn-d, Zn-p orbitals.

While in SnSe (Fig 1(b)), every Sn atom with six Se atoms bonding octahedral formation, and different layers along (001) direction are also made up of Sn-Se covalent bonds. The energy band of SnSe shown in Fig 2(b) illustrates that SnSe has an indirect band gap with the CBM and VBM at different positions in the Brillouin zone. The indirect gap value is 0.88*eV*, which is far less than that of ZnSe. From the partial density of states, we can know the CBM is contributed by Se(p) and Sn(p) electrons, and the VBM is contributed by Se(p) electrons. The band gap values calculated in this paper are in agreement with other theoretical calculation [14, 19, 20, 26] and experimental results obtained from optical measurements [4, 32].

**Fig 3:**
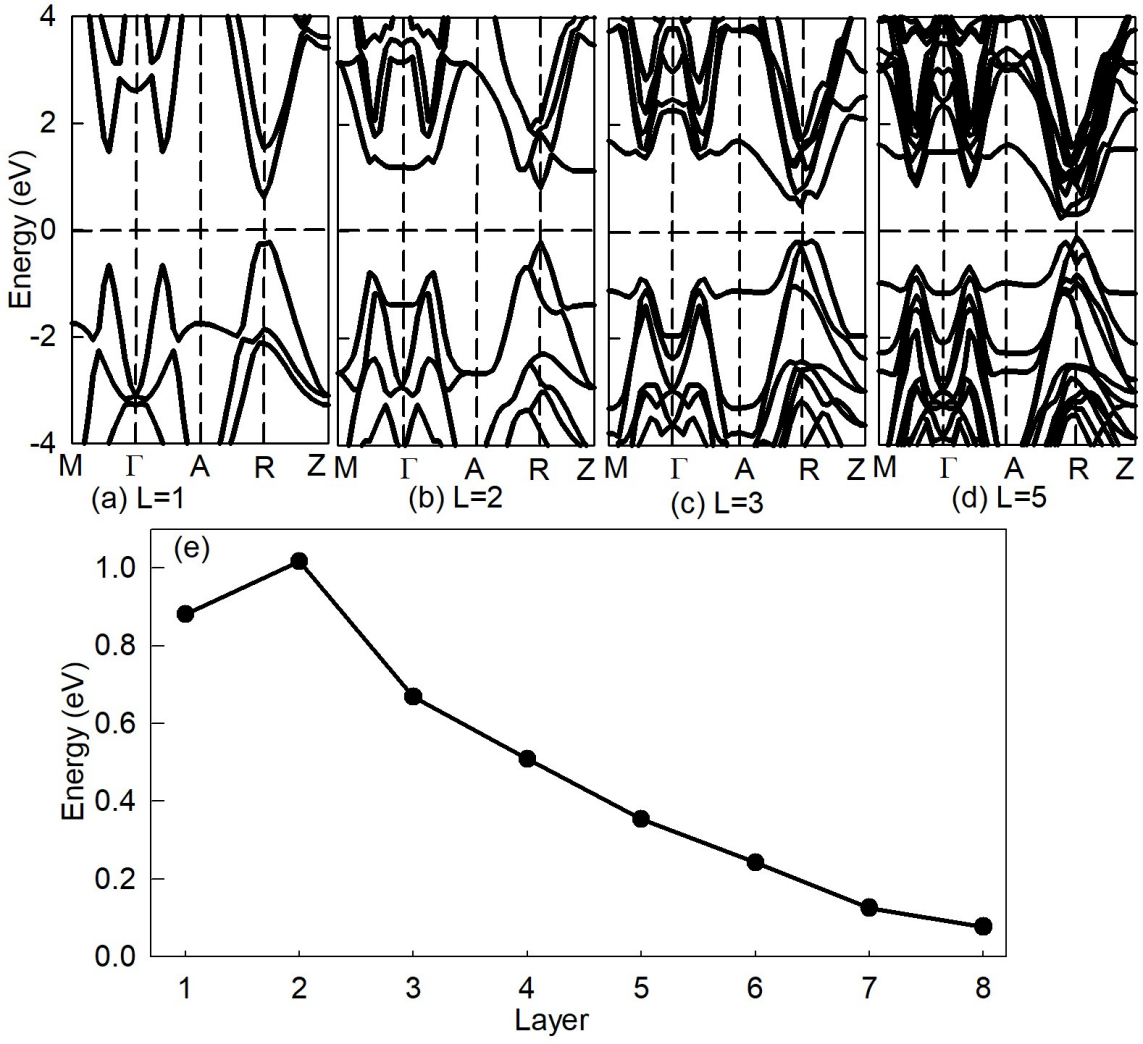
(a)∼(d) The energy band of SnSe with thickness of L = 1, 2, 3 and 5, respectively. (e) The relationship of band gap with the thickness of SnSe nanolayer.

Considering band gaps of nanocrystals can be regulated by changing the size materials, we suppose that the layer thickness of SnSe has great influence on electronic properties. Fig 3 shows the energy band structure of SnSe with different thickness of nanofilm. In general, the band structures of thin film materials with different layers have almost the same distribution. While with the increase of film thickness, the CBM moves to lower energy result in the band gap decreases gradually, meanwhile the overlapping hybridization between bands is enhanced and the band structure is more dense, which indicates that the quantum size effect decreases with the increase of film layer. That is to say the thinner the thickness, the more obvious the quantum size effect.

### Geometric structure of ZnSe/SnSe heterostructures

**Fig 4:**
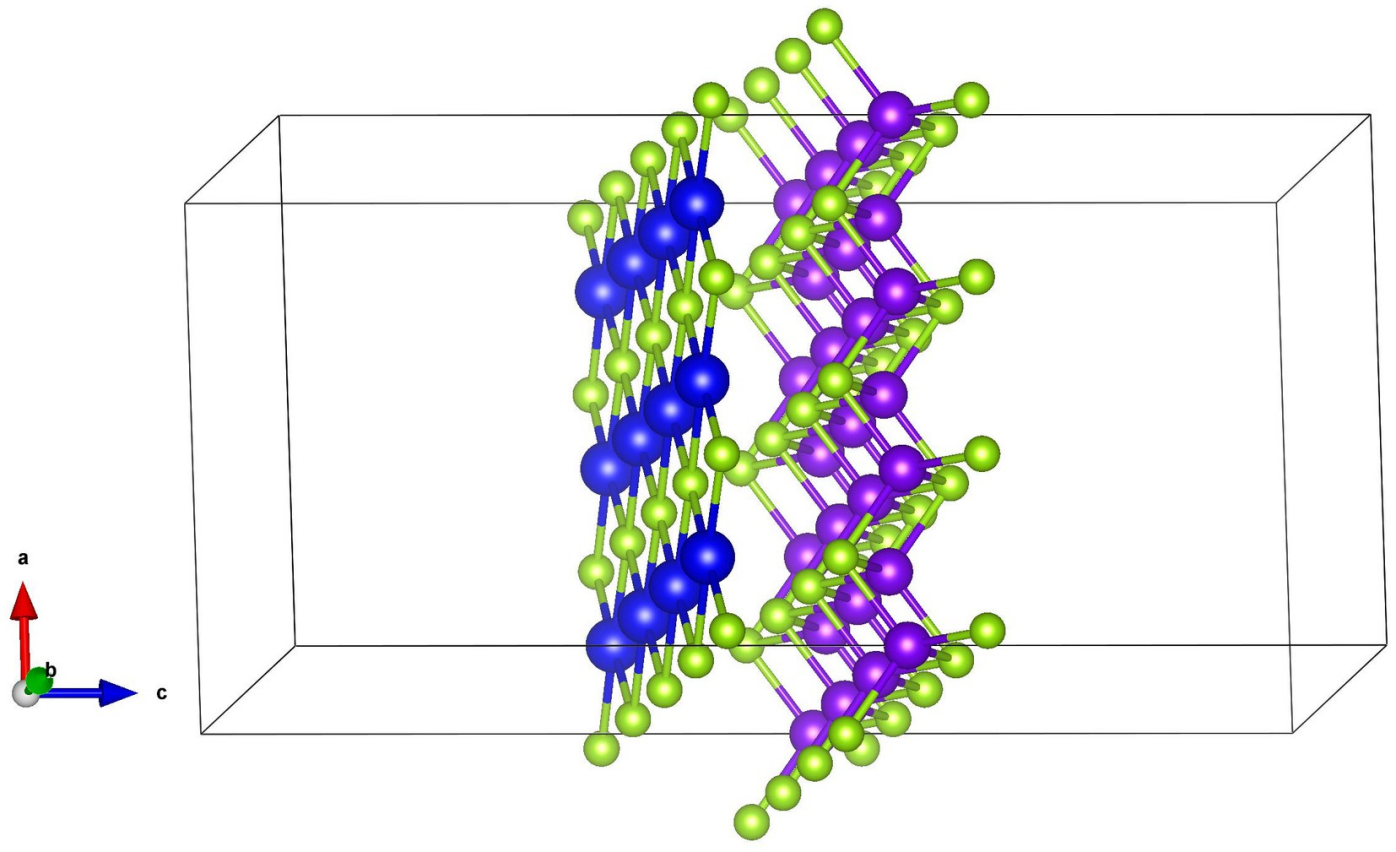
The structure of monolayer ZnSe/SnSe heterostructure from side view. The two ends of the material are vacuum layers with the distance of 20*Å*.

Although the two-dimensional materials ZnSe and SnSe have excellent and unique photoelectric properties, they also have some defects more or less, which limit their practical application. In order to expand their application range, we try to stack them together to form multi-layer heterostructure. Studies have shown that this type of heterojunction can greatly change the band alignment of the material, which in turn determines the properties of the material and its wider application [33, 34]. Due to the weak van der Waals bonding between ZnSe layers and the strong covalent bonding between SnSe layers, we constructed a heterojunction material with single-layer ZnSe and SnSe thin films, the ZnSe/SnSe heterojunction super-lattice is illustrated in Fig 4. Vacuum layer is arranged on the edge of materials with the distance of 20*Å*, which means that the distance between outermost atoms of adjacent films is 20*Å*, so the interaction between them can be neglected as well. Due to the lattice constants of two materials are quite different, lattice mismatch was introduced to simulate the stability of heterostructure. The interlayer lattice mismatch is 2.4%, which is quite small that the heterostructure can exist stably. To ensure the accuracy and stability of ZnSe/SnSe heterostructure, the interfacial binding energy was defined as follows:

$$\begin{array}{c} E=E_{ZnSe/SnSe}-E_{ZnSe}-E_{SnSe}, \end{array}$$ (1)

where *E*_*ZnSe*/*SnSe*_, *E*_*ZnSe*_, and *E*_*SnSe*_ are the total energy of ZnSe/SnSe heterostructure, monolayer ZnSe and N-layer SnSe, respectively. Results are listed in Table 1. When *E* < 0 means that the binding process will release heat, resulting in a stable interface, and the lower the binding energy, the more stable the structure. Notably, all the energies of different layers heterostructure are negative indicating the stability of ZnSe/SnSe heterostructures. As a result, five-layer heterojunction is the most stable, and the eight-layer heterojunction comes to the second, while the stability of two-layer system is the weakest.

**Table 1:** Interfacial binding energy of ZnSe/SnSe heterostructures with different layer of SnSe.

Layer	1	2	3	4	5	6	7	8
Energy (eV)	−0.4899	−0.3188	−0.4069	−0.5166	−0.8683	−0.5449	−0.6544	−0.7197

**Fig 5:**
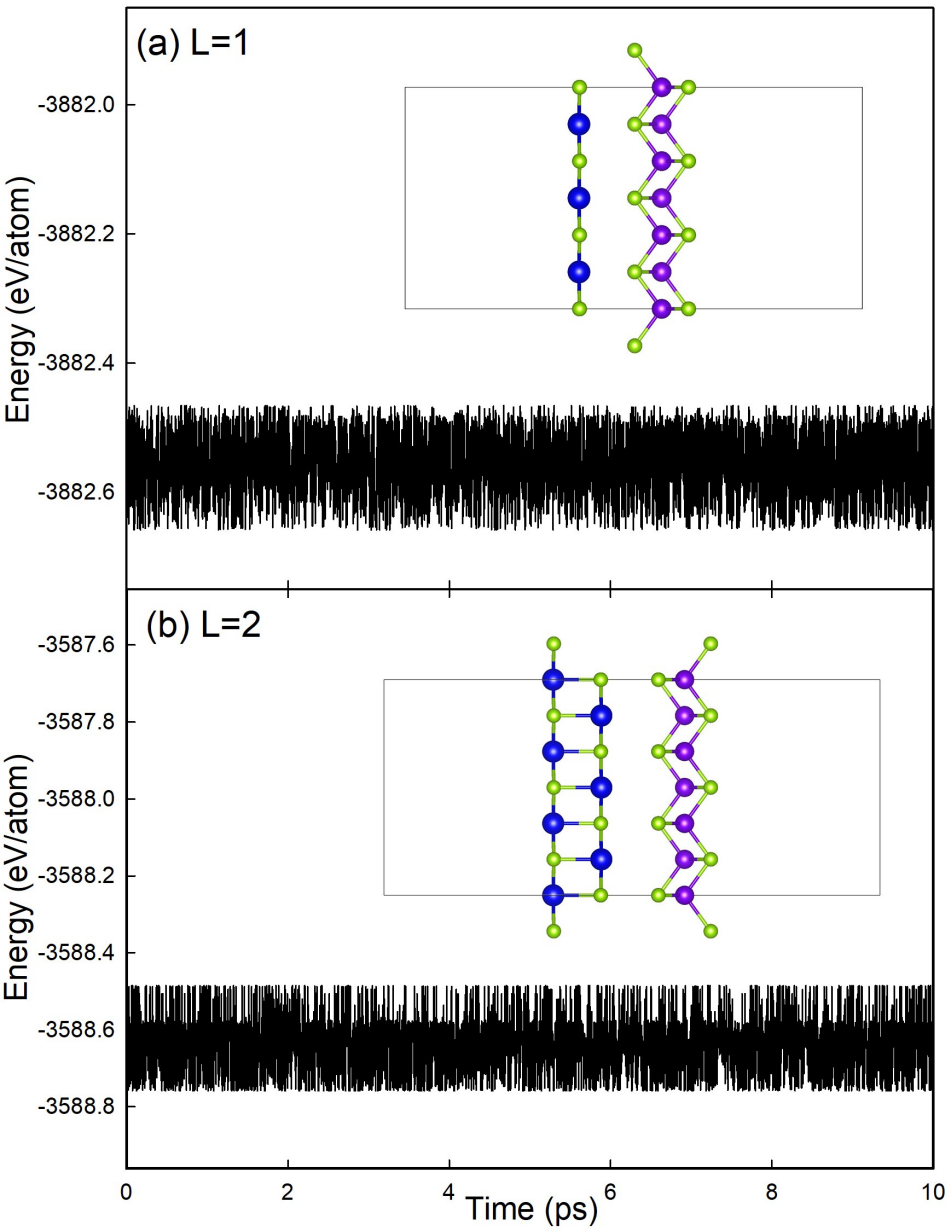
The free energy respect to time in AIMD simulation at 300K. (a) single-layer ZnSe/SnSe heterosture with 3 × 3 superlattice, (b) two-layer ZnSe/SnSe heterosture with 2 × 2 superlattice. The illustration is the corresponding structure of heterosture.

In order to describe the dynamic stability of ZnSe/SnSe heterojunction, the Ab initio molecular dynamics (AIMD) simulation [35] has been carried out. Here we set the temperature with 300K and the time step of 0.0025*ps*. The energy fluctuation of 3 × 3 and 2 × 2 superlattice of single-layer and two-layer heterostructures within 10*ps*, respectively. Results in Fig 5 shows that the energy fluctuates within a very small range and the material remains original structure, indicating the dynamic stability of ZnSe/SnSe heterogeneous.

**Fig 6:**
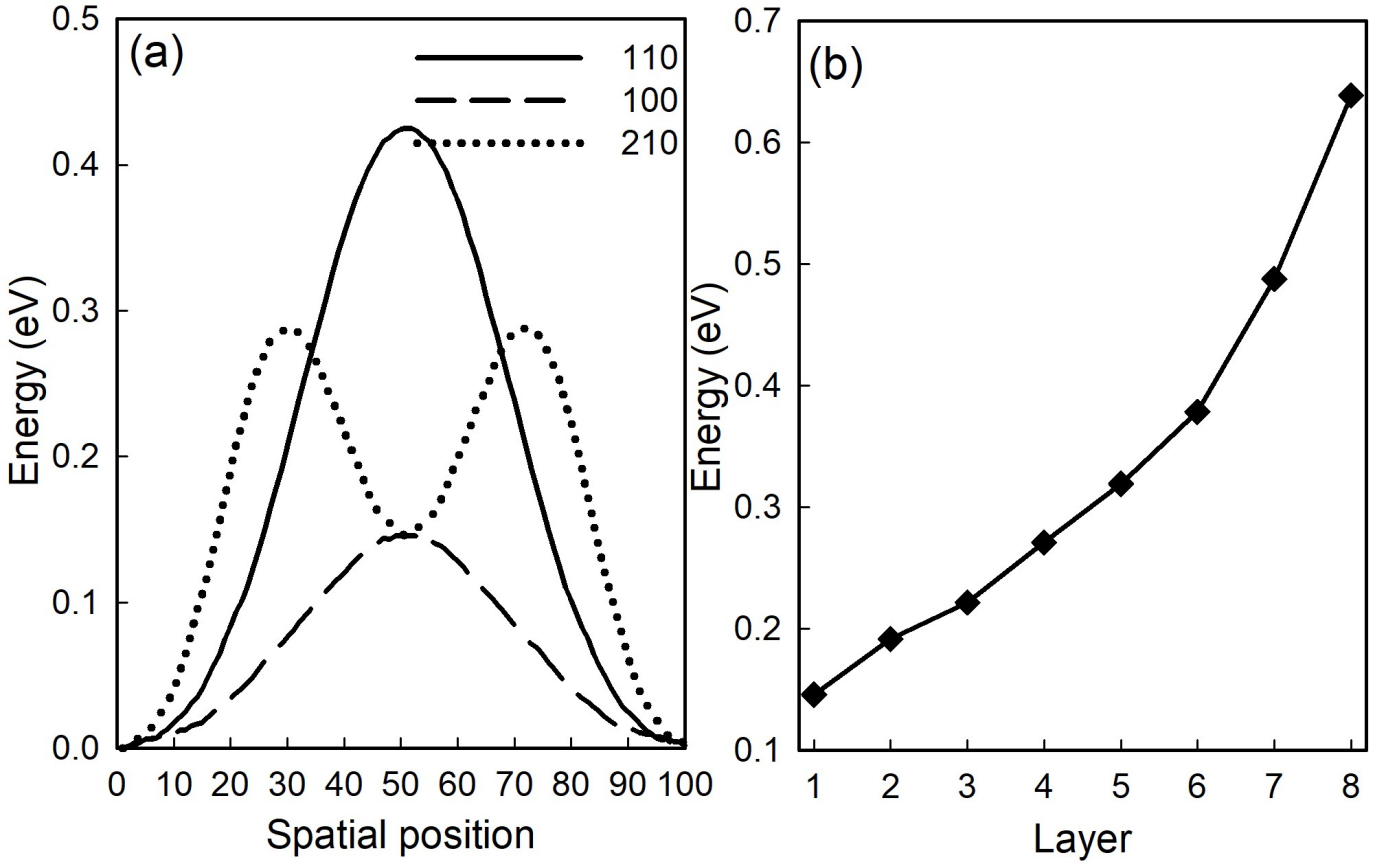
(a) Potential energy along different crystalline (solid line-(110); dashed line-(100); dotted-(210)) of ZnSe/SnSe heterostructure; (b) The minimun potential energy of ZnSe/SnSe heterostructure with different layer.

As for thin film materials, interlayer slip is one of the main cause of structural instability. The slip potential energy is the sum of interaction potential energy of all atoms at the film stacking in the unit cell, which can be used to describe the stability of the stacking structure. The results of potential energy along different crystalline were sheon in Fig 6. The greater the slip potential energy, the higher energy required for the lattice distortion caused by the slip dislocation of the material, and the less likely the material is to slip in this crystal orientation. From Fig 6(a) we can see that the potential energy has maximum value along (110)(solid line) compared with (100) (dashed line) and (210) (dotted line), so the ZnSe/SnSe heterostructure is more stable in this direction. As to (100) orientation, the potential barrier is about 0.15*eV*, while it is the minimum barrier of (210) crystal oriention,whose highest barrier is 0.3*eV*. The value and variation tendency of potention energy indicates that the material is more possible to slip along the direction of (210), and the required energy is 0.15-0.3*eV*. The minimum potential energy of ZnSe/SnSe heterostructure with different layer shown in Fig 6(b). The energy increases significantly with the increase of the number of layers. By comparing these results with interfacial binding energy listed in Table 1, we are pretty sure that only small amount of slips may happen between neighboring layers, while this little slip can’t make essentially change in structure of compound.

### Electronic properties of ZnSe/SnSe heterostructures

**Fig 7:**
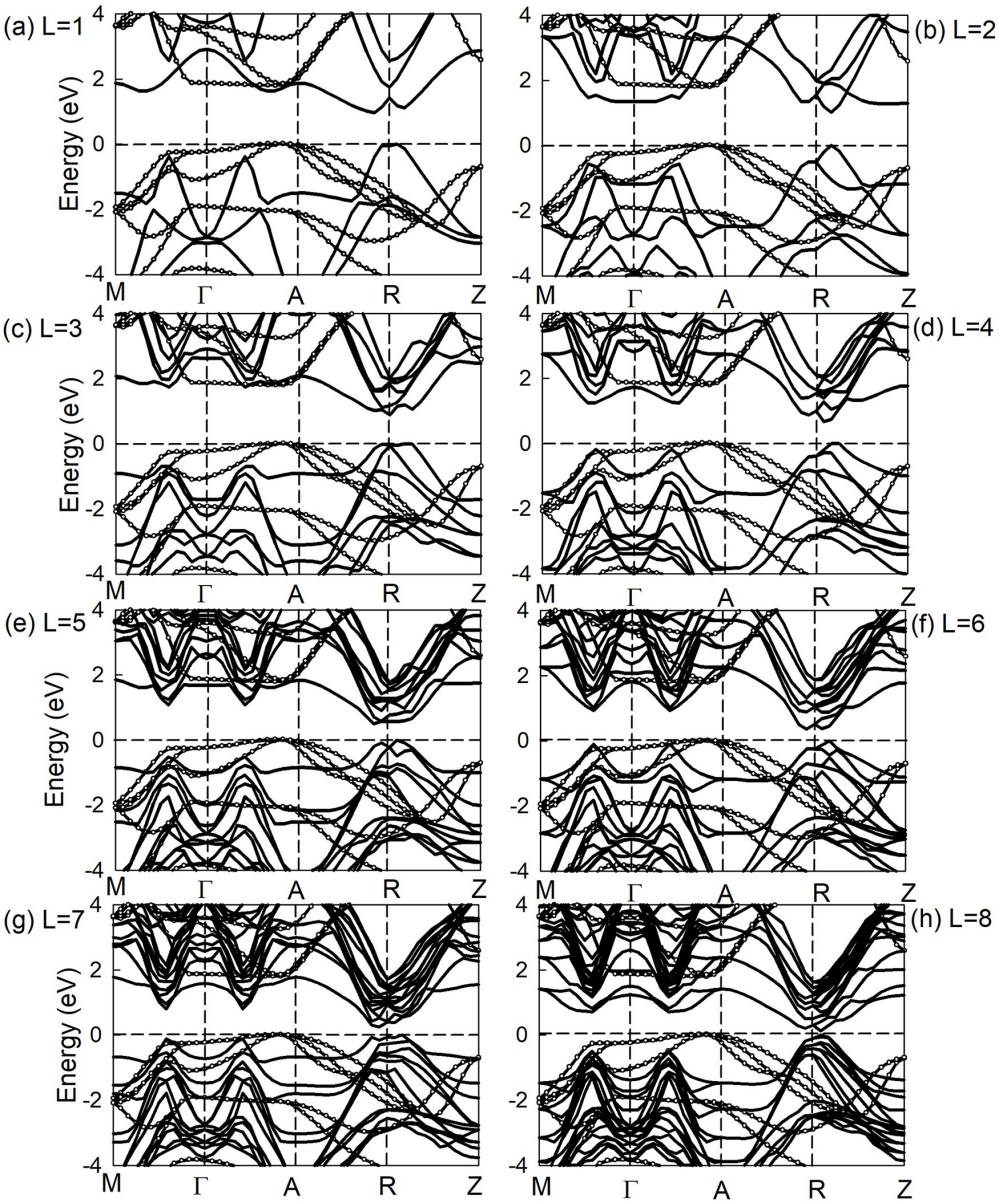
The band structure of ZnSe/SnSe heterostructure.

The band structures of ZnSe/SnSe heterostructure with different layers are shown in Fig 7. The solid lines are the contribution of SnSe films, and the dashed line with white circle means the donation from monolayer ZnSe. Generally speaking, the band structure of heterojunction is the superposition of the two pure thin film materials, which implied that the heterojunction retains electronic structural characters of two kinds materials independently. The valence band maximum (VBM) of heterojunction is basically located on the path of *Γ* − *A*, which is mainly contributed by the ZnSe material. While the conduction band minimum (CBM) of ZnSe/SnSe heterojunction is located on the G-R path, mainly from the SnSe film. With the increase of SnSe thin film layers, the conduction band bottom gradually moves to the vicinity of the Fermi surface, which leads to the decrease of band gap value of ZnSe/SnSe heterojunction.

**Fig 8:**
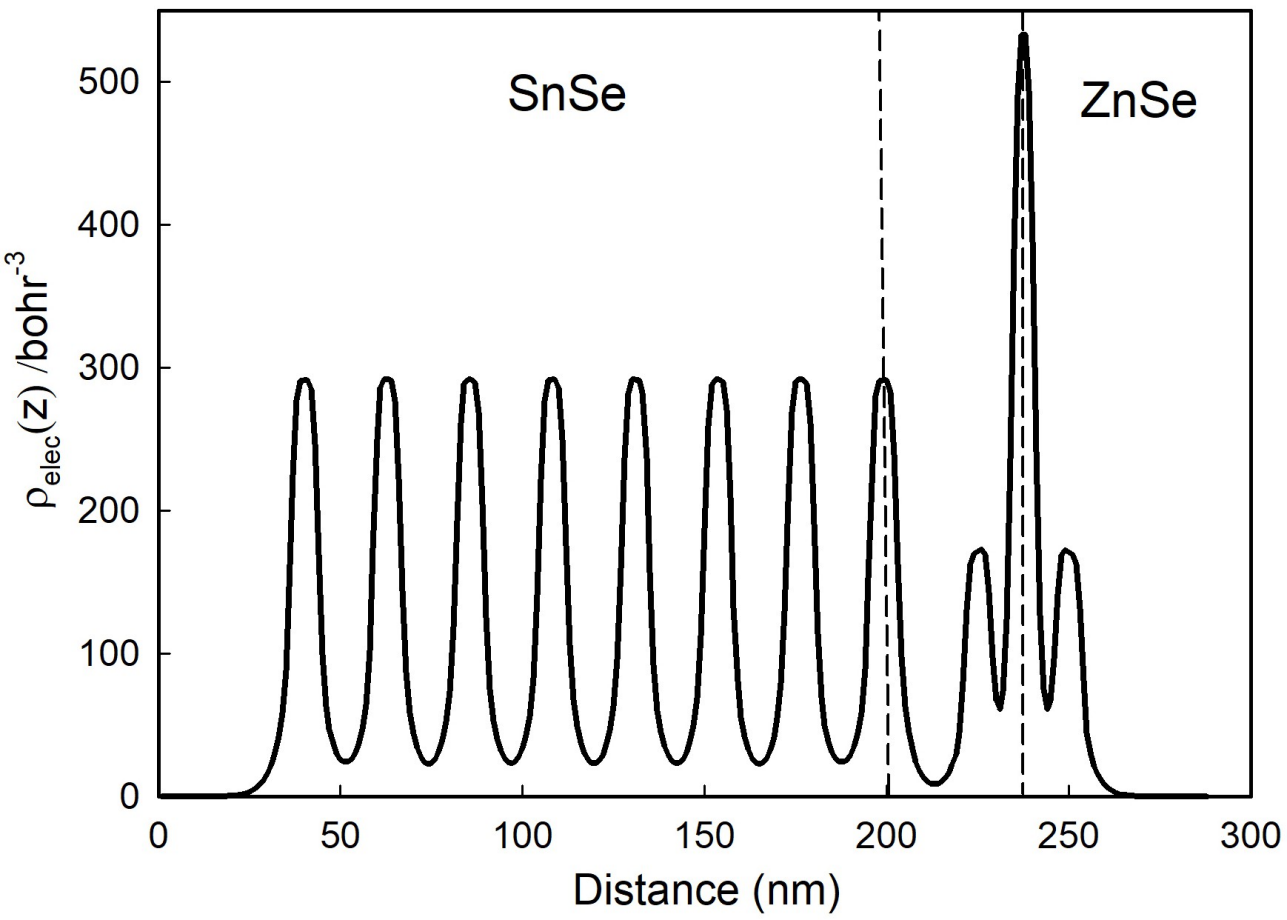
The electron concentration in real space along z-direction of 8-layer ZnSe/SnSe heterostructure. The dotted line range is the interface area.

Fig 7 also shows that CBM_*ZnSe*_ >CBM_*SnSe*_ >VBM_*ZnSe*_ >VBM_*SnSe*_, so the ZnSe/SnSe heterostructure is a typical Type-II material. This band structure will effectively promotes the separation of electron-hole pairs, so the material is often used in the fields of photoelectric detection, photocatalysis and solar cells. To illustrate detailed character of hole and electron transferring between interface materials, the electron concentration in real space along Z-direction *ρ*_*elec*_(*z*) is plotted in Fig 8.

*ρ*_*elec*_(*z*) provides physical picture of chemical bonds in the material, and it can be calculated as:

$$\begin{array}{c} \rho_{elec}\left(z\right)=2\int_{0}^{1}\int_{0}^{1}\sum_{i}\psi_{i}^{*}\left(r\right)\psi_{i}\left(r\right)dxdy, \end{array}$$ (2)

The value of *ρ*_*elec*_(*z*) highly depend on electrons distribution [8, 12]. From Fig 8 we can conclude that the same variation tendency in different layers of SnSe implied the interlayer atomic interaction of the chemical bond is much smaller than that of the intralayer atoms. The *ρ*_*elec*_(*z*) of SnSe decreases and the value of ZnSe increases at the interface, which resulting in the separation of hole-electron pairs in two different compounds, which will lead to the electric potential rise. That is to say, the variation of *ρ*_*elec*_(*z*) between ZnSe and SnSe interface (the dashed line area) indicates that the heterojunction can effectively promote the separation of electron-hole pairs and regulate the movement of carriers.

**Fig 9:**
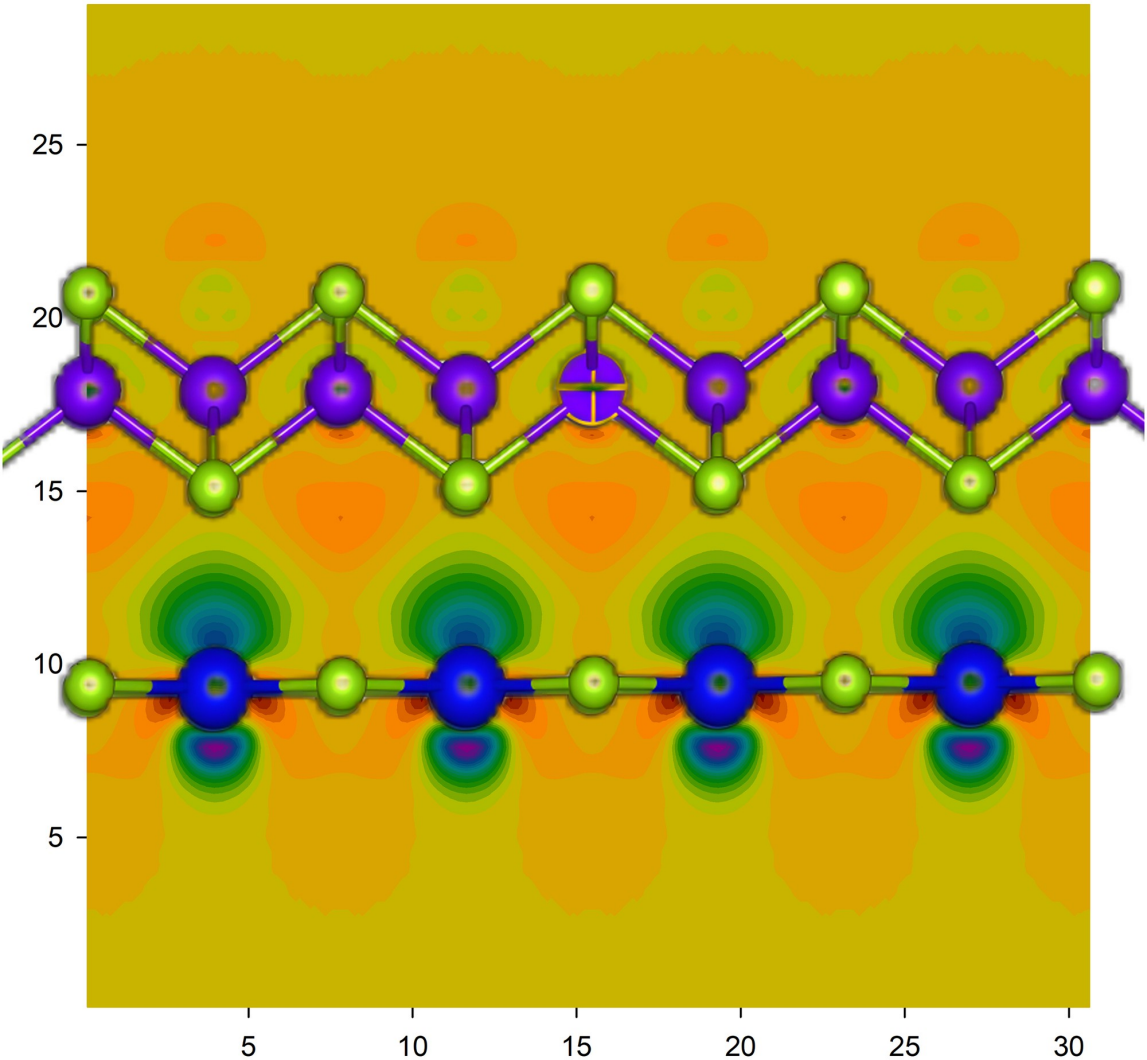
The charge density difference (Δ*ρ*) of ZnSe/SnSe heterostructure. The brown represents Δ*ρ* < 0 and the green is Δ*ρ* > 0.

With the construction of heterojunction, symmetry of original two-dimensional layered structure was destroyed, and charges will redistribute near the interface, along with the transfer of electrons between two monolayer materials of ZnSe and SnSe. Charge density difference (Δ*ρ*) defined by the following equation will describe the behavior of charge transport in ZnSe/SnSe heterostructure:

$$\begin{array}{c} \Delta \rho =\rho_{het.}-\rho_{ZnSe}-\rho_{SnSe}, \end{array}$$ (3)

where *ρ*_*het*._, *ρ*_*ZnSe*_ and *ρ*_*SnSe*_ are the charge density of ZnSe/SnSe heterostructure, monolayer ZnSe and SnSe film, respectively. And that Δ*ρ* > 0 represents charge accumulation and Δ*ρ* < 0 means charge depletion. In Fig 9, brown is negative and green is positive. The charge distribution in the layer is consistent with the atomic structure, in other words the charge is mainly concentrated around the atoms, which implies the covant bonding character in each single material. It can be seen from Fig 9 that there is a certain rule of charge depletion and accumulation. At the interface, charge depletion on the side of SnSe and a large amount of charge accumulation around monolayer ZnSe, so we can conclude that the electrons transfer from film of SnSe to monolayer ZnSe in ZnSe/SnSe heterostructure.

**Fig 10:**
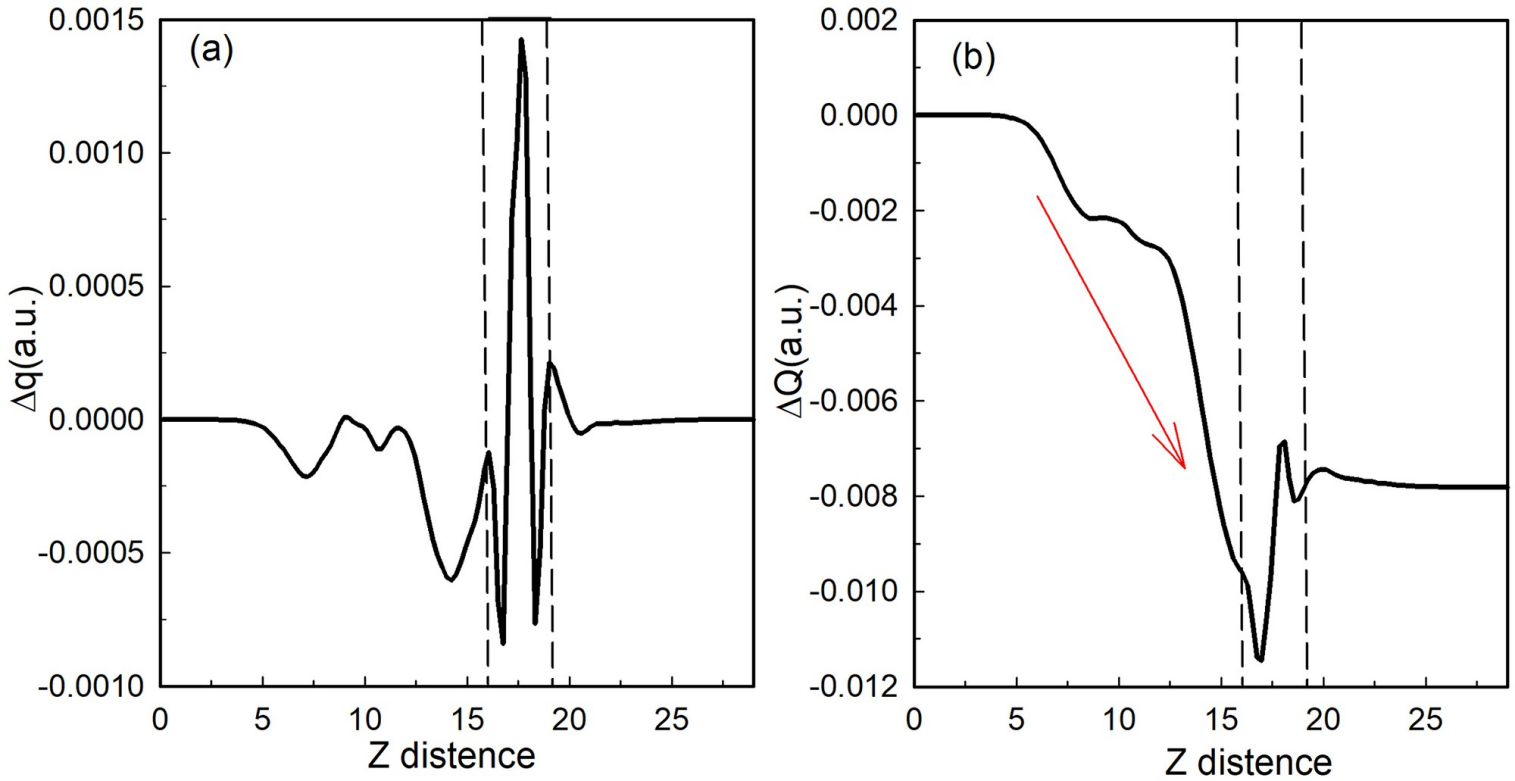
Charge displacement curve (CDC) (a)Δ*q* and (b) Δ*Q* of ZnSe/SnSe interface. The dashed line area represents the interface and the red arrow means the direction of electron moving.

In order to observe the interface charge transport characteristics more directly, we integrate the differential charge density (Δ*ρ*) both in the xy plane and z direction, and then calculate the charge displacement curve (CDC) with following equations [36]:

$$\begin{array}{c} \Delta q=\int dy\int \Delta \rho dx, \end{array}$$ (4)

$$\begin{array}{c} \Delta Q=\int \Delta qdx, \end{array}$$ (5)

where the area with Δ*q* > 0 means electron aggregation and the surface is negatively charged, otherwise (Δ *q* < 0) the film is positively charged. Δ*Q* reflects the accumulation of transferred chargesin the heterojunction. Along the z-direction, if Δ*Q* increases first and then decreases to zero, it indicates that the charge is transferred in the opposite direction. On the contrary, if Δ*Q* decreases to negative and then increases, it indicates that the charge is transferred along the z-direction. As shown in Fig 10(a), Δ*q* of monolayer ZnSe is positive and larger than that of SnSe, which is mostly negative, suggesting that SnSe can be a donor and ZnSe can be acceptor. Furthermore, result of Δ*Q* is mostly negatively, and in the interface region it decreases first and then increases, so electrons transport from SnSe to monolayer ZnSe, consistent with previous results of band structure and charge density.

In general, ZnSe/SnSe heterostructure is a Van Der Waals material with Type-II band structure. And as the thickness increases, the band gap narrows along with the interface effect weakens. Charge transfer analyses indicate that electrons transport from SnSe to monolayer ZnSe. Therefore, ZnSe/SnSe heterostructure can be a good candicate for two-dimensional solar cell material.

### Optical properties of ZnSe/SnSe heterostructures

Light absorption ability is an important rule in the application of photoelectric heterostructures. The absorption spectrum is calculated from dielectric function with the equation as follows:

$$\begin{array}{c} I\left(\omega \right)=\sqrt{2}\omega {\left[\sqrt{\epsilon_{1}^{2}\left(\omega \right)+\epsilon_{2}^{2}\left(\omega \right)}-\epsilon_{1}\left(\omega \right)\right]}^{1/2}, \end{array}$$ (6)

where *ω* is the frequency of energy, *I*(*ω*) stands for the absorption coefficient, *ϵ*_1_(*ω*) and *ϵ*_2_(*ω*) are the real and imaginary parts of dielectric function, respectively.

**Fig 11:**
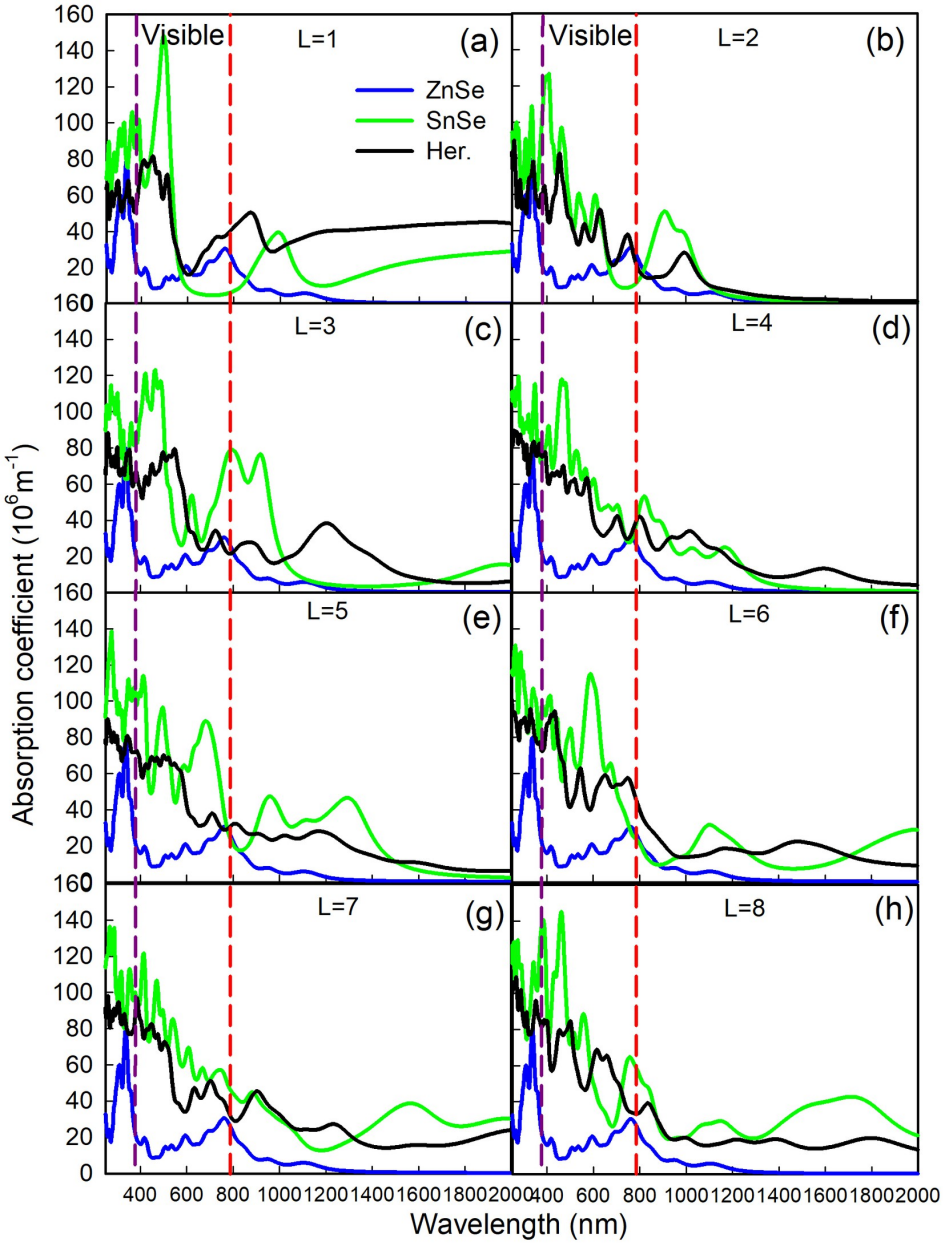
The absorption coefficients with different layers from *L* = 1 to *L* = 8. Blue, green, and black solid line represents the coefficient of ZnSe monolayer, SnSe interface, and ZnSe/SnSe heterostructure, respectively. The area between purple and red dashed lines stands for visible light. The left and right side of purple and red dashed lines is separately the ultraviolet and infrared spectroscopy.

The absorption spectrums of ZnSe/SnSe heterostructures, monolayer ZnSe and SnSe films with different layers are shown in Fig 11. All the heterostructures show better capacity to absorb light within the visible and infrared regions compared with ZnSe monolayer and SnSe films. In the visible light region, the high absorption coefficient peaks locate at about 390*nm*∼550*nm* of their respective spectrum range from 80 to 120(10^6^*m*^−1^). Besides, the ZnSe/SnSe heterostructures also show excellent absorption ability in near infrared spectroscopy with wavelength approximately 820*nm*∼1000 *nm*. All these regions covering most wavelength of sunlight arriving at earth [37, 38]. As to monolayer ZnSe, light absorption mainly occurs in shortwave region (λ < 1200*nm*), so the SnSe film plays an important role in optical properties and it can effectively improve the light absorption of ZnSe/SnSe heterostructures.

It’s obvious that all heterostructures have high absorption coefficients with wavelength range of 390*nm*∼1200 *nm*. Therefore, we can conclude that all the heterostructures are promising candidates for photoelectric vonversion applications. The maximum absorption peak of heterostructures was about 400*nm* with peak value almost 10^8^*m*^−1^. From Fig 11 we can see that with the increase of thickness, a red shift is produced and the spectral properties of heterostructure are enhanced as well. These phenomena are consistent with the tendency of energy band discussed in Fig 7. Under the influence of quantum size effect, increasing the thickness results in the reduction of relative surface area, then lead to the weakness of quantum size effect and strengthen of optical properties of ZnSe/SnSe heterostructures.

## Conclusion

In summary, based on first principles method we conducted a comprehensive study on the structural, electronic, and optical properties of ZnSe/SnSe heterostructures with different layers. Firstly, the structure and energy band results of two kinds of materials show bonding characters between atoms and interlayers. Then the interfacial binding energy and electron concentration in real space *ρ*_*elec*_(*z*) are calculated to examine structural stability of ZnSe/SnSe heterostructure. In this process, charge density difference and charge displacement curve describe interface interactions and electron (hole) transport behavior of heterostructures. Finally, the optical properties implied that ZnSe/SnSe semiconductor heterostructures have excellent photoelectric performance, which can be applied in integrated optics and integrated circuits. Therefore, this work offers a valuable insight into the interfacial effect on ZnSe/SnSe heterostructures, and also provides theoretical references for designing high performance optoelectronic devices.
